# Proposal of a Risk Stratification Model for Recurrence After Excisional Treatment of High-Grade Cervical Intraepithelial Neoplasia (HG-CIN)

**DOI:** 10.3390/diagnostics15131585

**Published:** 2025-06-23

**Authors:** Francesco Cantatore, Nadia Agrillo, Alessandro Camussi, Lucrezia Colella, Massimo Origoni

**Affiliations:** Department of Gynecology & Obstetrics, Vita Salute San Raffaele University School of Medicine, IRCCS Ospedale San Raffaele, Via Olgettina 60, 20132 Milan, Italy; cantatore.francesco@hsr.it (F.C.); agrillo.nadia@hsr.it (N.A.); camussi.alessandro@hsr.it (A.C.); colella.lucrezia@hsr.it (L.C.)

**Keywords:** Human Papilloma Virus (HPV), cervical intraepithelial neoplasia (CIN), conization, LEEP, follow-up, recurrence, prognostic factors, NRL

## Abstract

**Background/Objectives:** Cervical Intraepithelial Neoplasia (CIN) is a significant risk factor for the development of invasive cancer, and the histological detection of High-Grade CIN (CIN2+) during screening generally indicates the need for surgical removal of the lesion; cervical conization is the current gold standard of treatment. The recurrence risk for disease is reported to be up to 30%, based on data in the literature. Follow-up protocols mainly rely on High-Risk Human Papillomavirus (hrHPV) detection at six months post-treatment; if negative, this is considered the test of cure. This approach assumes that all patients have an equal risk of disease recurrence, regardless of individual characteristics. The objective of this study was to evaluate the individual recurrence risk using a mathematical model, analyzing the weight of various parameters and their associations in terms of recurrence development. **Methods:** We retrospectively examined 428 patients treated for CIN2+ at San Raffaele Hospital in Milan between January 2010 and April 2019. Clinical and pathological data were recorded and correlated with disease recurrence; three different variables, known to behave as significant prognostic factors, were analyzed: hrHPV persistence, the surgical margin status, Neutrophil–Lymphocyte Ratio (NLR), along with their relative associations. Data were used to engineer a mathematical model for the identification of different risk classes, allowing for the risk stratification of cases. **Results:** Surgical margins status, hrHPV persistence, and a high NLR index were demonstrated to act as independent and significant risk factors for disease recurrence, and their different associations significantly correlated with different recurrence rates. The mathematical model identified eight classes of recurrence probability, with Odds Ratios (ORs) ranging from 7.48% to 69.4%. **Conclusions:** The developed mathematical model may allow risk stratification for recurrence in a hierarchical fashion, potentially supporting the tailored management of follow-up, and improving the current protocols. This study represents the first attempt to integrate these factors into a mathematical model for post-treatment risk stratification.

## 1. Introduction

High-Grade Cervical Intraepithelial Neoplasia (HG-CIN) is a precancerous condition of the cervix and has been widely recognized as a preinvasive lesion that can progress to invasive cervical cancer if not appropriately treated in a timely manner [1,2,3,4,5]. Cervical cancer is the fourth most common malignancy among women worldwide, with an estimated annual incidence of over 500,000 new cases and the number of deaths exceeding 300,000 per year [6,7,8,9]. The prevention, diagnosis, and early treatment of cervical precancerous lesions such as HG-CIN play a crucial role in reducing the incidence of this invasive disease [10,11].

Human Papilloma Virus (HPV) is consistently recognized as the main etiological agent of cervical cancer. Sexual transmission of the virus and its ability to penetrate and persist in the cells of the female genital tract are considered determinants for the development of intraepithelial lesions. High-risk HPVs, such as types 16 and 18, are responsible for most cases of HG-CIN and cervical cancer [12]. The introduction of screening programs primarily based on HPV testing and reflex cervical cytology (Pap test) have significantly improved the early diagnosis and treatment of precancerous lesions, reducing the rate of progression to invasive cancer [13,14]. The gold standard of treatment for HG-CIN is currently represented by conservative excisional procedures, namely cervical conization and the Loop Electrosurgical Excision Procedure (LEEP); however, despite the effectiveness of these conservative treatments [15,16,17], a significant percentage of the patients treated for HG-CIN develop recurrences by the postoperative follow-up, with the highest rates within the first 24 months after treatment. Recent studies report recurrence rates ranging from 5% to 30%, depending on the clinical–pathological characteristics of the patients and the follow-up methodology adopted [18,19,20,21]. Several factors have been identified as predictors of recurrence in surgically treated HG-CIN patients [22,23,24,25]. Among these, the status of surgical margins and the presence of post-treatment hrHPV positivity have been widely studied and recognized as highly significant determining factors [26,27,28,29,30]. The persistence of high-risk HPV infection is associated with a significant increase in the risk of recurrence, while positive surgical margins are suggestive of an incomplete resection of the lesion, contributing to the possible recurrence of the neoplasm [19,31,32,33,34]. For these reasons, hrHPV positivity after treatment, having demonstrated a high Positive Predictive Value (PPV) for recurrence, is considered the test of cure. Another prognostic factor that recently emerged as a possible marker of recurrence is the Neutrophil/Lymphocyte Ratio (NLR) index, a systemic marker of inflammation [35]. Chronic inflammation is recognized as a contributory factor in the development and progression of neoplasms, and an altered neutrophil–lymphocyte ratio has been linked to poor prognosis in several neoplasms. Since it is associated with an increased risk of recurrence in patients with HG-CIN, recent studies have suggested that an elevated NLR may also have value in preinvasive cervical conditions, providing a potential non-invasive prognostic tool to identify those patients at higher risk [36,37,38,39,40]. Currently, the follow-up protocols after conservative treatments are solely based on hrHPV testing at six months after conization or LEEP, thus considering all treated patients at the same risk for recurrence, independently of other clinical–pathological features.

The aim of this study was to identify the major negative prognostic factors associated with the recurrence of high-grade cervical intraepithelial neoplasia in patients undergoing conservative treatments, alone or in association with one another. In detail, we examined the relative influence and associations of known variables such as the surgical margin status, post-treatment HPV DNA detection, and the Neutrophil–Lymphocyte Ratio (NLR) on the risk of recurrence, with the aim of developing a risk stratification model that would support clinicians in managing patient follow-up. A risk stratification model based on these clinical variables, in our opinion, could help to improve the effectiveness of follow-up, allowing for the personalization of patient surveillance, identifying subgroups at different recurrence risk, and allowing for a reduction in unnecessary invasive interventions for those at low risk. Furthermore, a better understanding of the role of systemic inflammation, measured using the NLR, could provide new perspectives for therapeutic approaches in patients with HG-CIN, offering the opportunity to integrate strategies based on the modulation of inflammation.

## 2. Materials and Methods

**Study Group.** This study retrospectively reviewed 623 patients treated for HG-CIN at San Raffaele Hospital in Milan between January 2010 and April 2019. Patients underwent conservative excisional treatments (cervical conization or LEEP procedures) for CIN2, CIN3, and carcinoma in situ, detected through the Italian cervical cancer screening program. Demographic, histological, surgical, and clinicopathological data, pre- and post-treatment HPV DNA status, and follow-up information up to June 2020 were collected. Patients under immunosuppressive drugs regimens (e.g., organ transplant cases) or affected by immune system impairments (e.g., HIV positive cases), were excluded from the study. Excisional procedures were performed by experienced colposcopists, while preoperative and follow-up pathological analyses were conducted by trained pathologists from the same institution. Recurrence was defined as a new diagnosis of CIN1+ during follow-up. Conization was performed as a one-day surgery procedure, and the NLR was recorded preoperatively. A minimum of 24 months follow-up with adherence to the standard protocols and the availability of clinical/pathological records were required for inclusion of the cases in the study.

**Variables analyzed.** According to the existing literature data [18,21,36,41,42,43,44], the surgical margin status of the excised cervical specimen, hrHPV-DNA detection at the first follow-up visit, and an NLR index with an established cut-off value of 2.0 obtained through a blood sample taken on the same day as the surgical procedure, were considered in the study analysis and correlated with disease recurrence; this latter event was defined as a histologically proven CIN1+ lesion during follow-up.

**Study Design.** In relation to investigating the effectiveness of the proposed mathematical model, these variables were chosen according to their independent statistically significant value in predicting recurrences after conservative cervical treatment. Since the scope of the study included a preliminary evaluation of the potential utility of the model in clinical practice, we intentionally limited the number of variables submitted for investigation, selecting parameters which already have an independent prognostic significance; in our view, this design better fits the scope for validating the model by initially stratifying the risk for known variables only.

**Ethical Issues.** In accordance with the guidelines, internal Institutional Review Board (IRB) approval was obtained after submission to Ethics Committee for evaluation (protocol n. 84/INT/2020).

**Mathematical Methodology.** Regarding the mathematical methodology adopted, each single prognostic factor was first analyzed using a univariate logistic regression correlated with the prevalence of recurrence and statistically weighted; in detail, the dataset consisted of 428 instances (i.e., patients), three attributes (or predictors): A_1_ (hrHPV), A_2_ (surgical margins), and A_3_ (NLR), and a label for the probability of recurrence R (1 for positive vs. 0 for negative, in the dataset). Secondly, to further investigate the extent of the interactions among the risk factors, four binary variables (NLR+Margins, Margins+hrHPV, hrHPV+NLR, NLR+hrHPV+Margins) were created and similarly analyzed by labelling them as 1 if all the variables considered were present in each subject, and as 0 if not. Univariate logistic regression analysis was conducted to measure the association between these new variables and CIN recurrence, and to measure the weight of the variables’ associations. All individual and combined variables were then evaluated using a decision tree analysis to segment the patients into different groups based on their risk of recurrence, thus establishing a hierarchical order of the variables’ importance. The purpose of the latter statistical analysis was to train the predictive model to evaluate the recurrence probability of a patient using the known values of the corresponding three predictors.

A non-linear, multivariate regression model was elaborated, according to the mathematical principles of mixed linear–nonlinear least squares regression [45], using the following formula:

R (A_1_, A_2_, A_3_) = C_0_ + C_1_A_1_ + C_2_A_2_ + C_3_A_3_ + C_4_A_1_^2^ + C_5_A_2_^2^ + C_6_A_3_^2^ + C_7_A_1_A_2_ + C_8_A_1_A_3_ + C_9_A_2_A_3_

where the C coefficients are generated by the regression model itself, depending on the dataset variables values. Once the C coefficients were identified from the fitting, the formula was used to determine the R values (i.e., the probability of recurrence) for the different associations of the three predictors A_1_, A_2,_ and A_3_ for each patient; in our specific case, the three predictors only had a discrete value of 1 or 0, leading to a total of eight possible input combinations for the variables (Table 1).

Consequently, the predictive model defined above has been used to estimate the probability of recurrence (R) associated with each of these combinations. By sorting the eight combinations according to the corresponding predicted value of R, the risk stratification model was effectively produced.

**Statistics.** Statistical analysis was performed using the package version 20.0 for Windows (SPSS). All statistical tests were two-sided, and significance was established at a *p*-value < 0.05.

## 3. Results

Of the entire cohort of 623 patients treated during the interval analysis and considered as eligible, a total of 428 cases fulfilled the inclusion criteria and were included in the study, while 195 were excluded (160 were incompletely followed up according to the study parameters and 35 were lacking complete clinical records). These 428 cases represented the dataset used in the engineering of the mathematical model, using a step-by-step statistical process. The mean and median age of the patients were 38.4 and 37.5 years, respectively. All patients, in accordance with the internal institutional guidelines of clinical management, were submitted for LEEP excisional one-day surgery procedures; according to surgical specimen histology, a high-grade squamous intraepithelial neoplasia (HG-SIL) (CIN2, CIN3, and carcinoma in situ) was recorded in 90.4% of cases, while a low-grade squamous intraepithelial neoplasia (LG-CIN) (CIN1) was recorded in 9.1% of cases; a high-grade glandular intraepithelial neoplasia (HG-GCIN) (G-CIN2 and G-CIN3) was detected in 0.4% of the whole group. Surgical cone margins were positive in 15.2% of cases and negative in 84.8%. Postoperative follow-up hrHPV testing was positive in 27% of cases and negative in 73%. The NLR value was <2.0 in 256 (59.8%) patients and ≥2.0 in 172 (40.2%) patients. The overall recurrence rate accounted for 20% (18.2% within 12 months from conization and 1.8% within 24 months, respectively). We considered the finding of CIN1+ at the biopsy performed at follow-up colposcopic controls as a recurrence. The study cohort demographics and clinicopathological characteristics are summarized in Table 2.

After univariate logistic regression analysis, CIN recurrence was statistically associated with all variables, representing possible interactions among risk factors (*p* < 0.05). We obtained five different odds ratios (ORs), considering expression of all possible combinations of the three variables. In detail, the OR (CI 95%) for recurrence was 3.417 (1.733, 6.737) for NLR ≥ 2 + hrHPV persistence, 4.507 (2.006, 10.126) for NLR ≥ 2 + Margins, 6.913 (2.202, 21.705) for hrHPV persistence + Margins, 5.512 (1.210, 25.109) for NLR ≥ 2 + hrHPV persistence + Margins, and 0.243 (0.127, 0.463) for no risk factors (Table 3).

The obtained odds ratio values show a cumulative risk effect in relation to the different combinations of the three variables. The largest effect in terms of risk (OR 6.913) was obtained for the combination of hrHPV persistence and positive surgical margins, indicating a 7-fold increase in recurrence risk, compared to the protective effect against the same risk (OR 0.243) that was determined in those cases having none of the three variables considered, e.g., negative hrHPV testing, negative surgical margins, and an NLR < 2.

**Figure 1:**
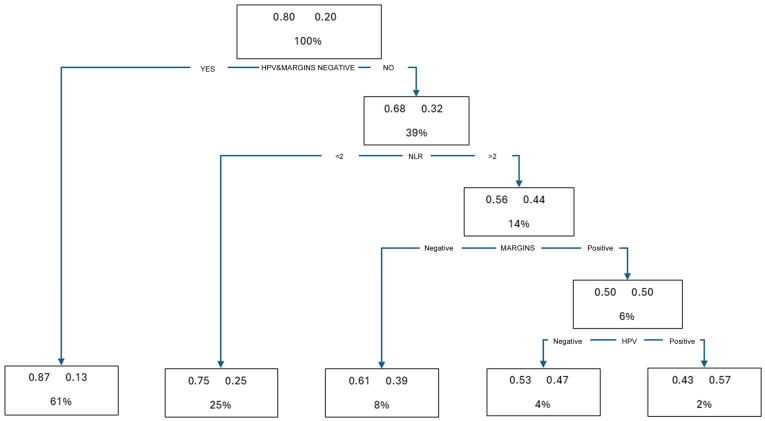
Decision tree model according to the significant variables.

A decision tree model that considered all the possible interactions among the variables, alone or in association, was then created by the mathematical model itself, identifying the statistical weight of how the recurrence risk increased according to the presence/association of the variable (Figure 1).

The chart defined five classes of risk according to their increasing recurrence rate. The first leaf at the apex of the graph includes the entire study group of patients (428 cases) in the dataset, with an overall 20% recurrence rate. The first branch identifies two subsets of patients: one including cases with negative hrHPV testing and negative surgical margins, and another subset in which hrHPV and/or surgical margins were positive; this latter subset of cases did not yet consider the NLR variable. The recurrence rates in these two groups were 13% and 32%, respectively. The latter subgroup was further analyzed, adding the variable NLR; two additional branches were obtained, correlating with the following recurrence rates: 25% in cases with an NLR < 2 and 44% in cases with an NLR ≥ 2. The following step was splitting the NLR ≥ 2 subset, which was further evaluated for the surgical margin status variable: recurrence rates were 39% and 50% in cases with negative and positive surgical margins, respectively. The final analysis was then performed for the positive surgical margins subset of cases, introducing the hrHPV persistence variable: the recurrence rate was 47% in cases with negative hrHPV testing, increasing to 57% when the hrHPV variable tested positive. Based on all the recurrence rates, a hierarchical order of decreasing recurrence risk was obtained, from the highest to the lowest, and a risk stratification model created, allowing the identification of eight classes at significantly different risks of recurrence according to the variables’ status; the recurrence risk ranking is summarized in Table 4.

The highest probability of recurrence was 69.4% and was recorded in cases with positivity for all three variables under study, while the lowest probability of recurrence (7.48%) was obtained for the group of patients without any of the variables considered. All possible combinations of the variables that, through different interactions, define an intermediate probability of recurrence have been stratified accordingly.

## 4. Discussion

More than 60% of cases of low-grade cervical intraepithelial neoplasia (CIN1) spontaneously regress to normal, while high-grade CIN is considered a lesion that can progress to invasive carcinoma if left untreated [1]. High-grade cervical intraepithelial neoplasia is a frequent precancerous condition in the female population, characterized by a relatively high potential for recurrence after conservative surgery [19,20].

However, not all patients undergoing conservative surgery for CIN2+ have an increased risk of recurrence or disease progression compared to untreated controls. Therefore, it is conceivable that a tailored and personalized follow-up approach, based on the individual recurrence risk, would allow for the careful surveillance of patients at high risk of recurrence and a less intensive follow-up for those at very low risk of recurrence; this approach would very likely result in a substantial reduction in the workload for follow-up visits and diagnostic procedures, and also decrease the amount of unnecessary procedures. The concept of assessing the real risk of a specific pathological condition, through the stratification of patients according to their individual characteristics, is currently widely supported and adopted in many circumstances, mainly focusing on a preventive perspective. In our opinion, based on the present experience, extending the application of risk assessment and patient stratification to post-treatment follow-up protocols represents a viable and promising option. Moreover, a stratification-based risk assessment would be essential for tailoring appropriate follow-up programs, especially in low-resource settings. In addition, a reduction in interventional needs during follow-up, secondary to the identification of patients at higher risk for recurrence after primary treatment, would potentially result in significant added benefits to the patients. It is known that, although a single correctly performed conization has a minimal impact on women’s fertility and obstetrical outcomes, multiple cervical excisions are significantly correlated with several obstetrical complications, such as the risk of preterm birth and the premature rupture of membranes [46,47].

In addition to the strongly validated factors for the risk of post-treatment recurrence, which include patient age at diagnosis, surgical cone margins [48,49], and high-risk Human Papilloma Virus status at follow-up [22,24,28,33], several other co-factors play a prognostic role in the development of disease recurrence and reportedly have significant value. In particular, personal habits regarding smoking, sexual behavior, oral contraceptive intake, pre-treatment severity of the lesion, and treatment modality have all demonstrated statistical associations with recurrence rate; however, none of these factors demonstrated an independent prognostic significance when submitted to multivariate analysis [18]. More recently, HPV type-specific genotyping, both pre- and post-treatment, demonstrated a higher Positive Predictive Value (PPV) compared to qualitative hrHPV detection for the assessment of recurrence risk [41,42,50]. The role of HPV vaccination, according to recently reported data, is of high interest in the scenario of cervical preinvasive lesions management: independently of the timing of HPV vaccine administration, either as primary prevention or as an adjuvant prophylaxis at the time of cervical conization, HPV vaccines have a favorable effect on post-treatment recurrence rates. However, a recent meta-analysis concluded that the strength of the available evidence is still low, and data must not yet be considered conclusive [51]. One of the most interesting and promising areas of investigation is represented by the recently discovered association between the vaginal microbiota and the natural history of HPV-related diseases, including the risk of recurrence after conservative treatment. Many data are currently available, reporting on the role of specific vaginal microbiota patterns, the so-called Community State Types (CSTs), in HPV infection and cervical intraepithelial neoplasia [52,53,54]; however, all the published reports are consistent in concluding that the vaginal microbiota assessment cannot yet be considered a clinically meaningful and practically useful tool. This conclusion has also been summarized in a recently published meta-analysis [55].

In terms of clinical applications, it has been reported that the combination of HPV positivity and positive surgical margins represents the most effective strategy for the identification of women at significantly higher risk of recurrent disease [30,34]. In line with the goal of identifying patients at higher risk, one study proposed the integration of several different variables that contribute to CIN recurrence, defining a score-based nomogram for the risk of recurrence after cervical conization treatment [56].

The Neutrophil–Lymphocyte Ratio is a novel hematological parameter for systemic inflammation and is widely adopted in almost every medical discipline as a reliable and easily available marker of the immunological status in response to infectious and non-infectious conditions. The NLR reflects the ongoing dynamic relationship between the innate (neutrophils) and adaptive (lymphocytes) cellular immune response during illness and in various pathological states, and a normal value in healthy individuals is <2 [57]. The clinical usefulness of the NLR assessment has been largely documented in solid tumors, demonstrating that preoperative NLR measurements have a significant prognostic significance in terms of their correlation with cancer extent, metastatic potential, lymph node involvement, response to therapy, and disease-free intervals [57]. The same data are available for gynecological cancers, and particularly cervical cancer [37,39,58]. Accordingly, we wanted to explore whether preoperative NLR detection could also be of use in pre-invasive cervical conditions, correlating with the recurrence rate after cervical conization for HG-CIN. We recently tested this hypothesis by generating a receiver operating characteristic (ROC) curve analysis and determining the Youden Index [21], identifying an optimal cut-off value of 2.0 for predicting recurrence; we then demonstrated a significant correlation between the Neutrophil–Lymphocyte Ratio (NLR) and the risk of HG-CIN recurrence, independently of the surgical margins and post-treatment hrHPV status [36]. With regard to the NLR cut-off value of ≥2.0 identified in our previous experience after generating the ROC curve [21], a meta-analysis by Ethier [39] involving 26 studies reporting on 10,530 cases of gynecological cancers identified that mean NLR cut-off values of 2.95 and 2.79 significantly correlated with overall survival and event-free survival, respectively. In line with our results, several other authors have investigated the prognostic significance of the NLR, together with other co-factors, in predicting recurrences after the conservative treatment of pre-invasive cervical conditions (CIN2+). Chun [59] studied 230 patients, reporting that the optimal NLR cut-off value for recurrence was 2.1 and that the Recurrence-Free Survival (RFS) was significantly lower in patients with an NLR > 2.1. The authors concluded that the NLR acts as an independent prognosticator of RFS in CIN individuals. Farzaneh [35] investigated 307 cases and found that an NLR cut-off value of 1.9 was the most appropriate for the prediction of recurrences; a higher NLR value (>1.9) was significantly associated with a higher recurrence rate and a lower RFS. Hajizadeh [60] studied the same cohort of 307 patients using a defective model and reported that an NLR cut-off value of >1.9 and a 15 mm^2^ cervical excision offered significant prognostic value; the authors demonstrated that cure rates ranged from 98% in cases with an NLR < 1.9 and excisions > 15 mm^2^, to 30% in patients with an NLR > 1.9 and excisions < 15 mm^2^. An Italian study by Dominoni [44] including 287 cases of CIN2+ and 157 cases of CIN1, reported a 14% recurrence rate and an optimal NLR cut-off value of 1.34; this apparent discrepancy with the majority of reported results is probably due to the higher number of CIN1 patients included in the study; these are known to correlate with very low recurrences rates, and this very likely influenced the NLR cut-off determination.

The present study reinforces the value of including the NLR as a prognostic factor in estimating the probability of recurrence during the follow-up of patients undergoing conservative excisional procedures for HG-CIN, together with follow-up hrHPV detection and surgical margins status. The relative weight of the interactions among the variables using a mathematical model has been evaluated, demonstrating that the simultaneous presence of multiple risk factors increases the risk of recurrence when compared to the risk associated with each single variable alone; additionally, the NLR was demonstrated to be an independent risk factor for recurrence after multivariate analysis. It was also evident from the statistical analysis that the risk of recurrence in the absence of the three risk factors is significantly reduced, and that this condition is protective; individuals with all-negative variables present a recurrence risk that is significantly lower compared to the risk correlated with each negative variable individually considered. The decision tree approach and the statistical analysis confirm that the relative impact of the NLR variable is, in hierarchical order, the least significant and, on the other hand, that the surgical margin status is the most relevant; our results are in line with Onuki [27] and Ghaem-Maghami [61], who reported similar recurrence risks for post-treatment CIN2+ in patients with positive surgical margins and positive hrHPV testing in the follow-up.

Our sensitive and high negative predictive value data are in line with the literature and demonstrate how the use of these variables can have a significant impact in the clinical management of follow-up.

The current Italian recommendations, together with many other international guidelines, indicate that co-testing (Pap test + hrHPV testing) six months after surgical treatment for HG-CIN [62,63], with or without a colposcopic evaluation, is acceptable; thereafter, patients enter a scheduled follow-up protocol for several years. In our view, a potential limitation of the current follow-up protocols may be the lack of risk assessment for patients entering post-treatment monitoring. The present study could represent a starting point for considering the hypothesis of stratifying patients in the follow-up, as already done in the preventive diagnostic workup [62]. One of the major advantages of this approach would be a potentially significant reduction in the number of colposcopies performed in post-treatment, particularly for those individuals stratified in the very low risk category. As an example, a pre-treatment NLR value < 2, together with negative surgical conization margins and negative hrHPV testing at first follow-up visit, may allow a prolonged interval between follow-up visits, or even an anticipation of the patients’ return to routine screening protocols. On the other hand, in young and nulliparous patients in whom a higher risk for recurrence is preoperatively identified, incomplete and/or insufficient cervical excisions could be more easily avoided. In our view, after confirmation of our data in similar studies, the application of the risk stratification principles in the follow-up of conservatively treated patients represents the natural evolution of the innovative strategies that have been widely accepted and adopted in many countries, targeted at cervical cancer prevention.

Despite the interesting and encouraging results, some limitations of the present study must be discussed. The retrospective nature of the study may represent a bias; however, almost all studies reporting on the prevalence of post-conization recurrences are based on retrospective analysis and the interpretation of historical data [51]. The second limitation can be attributed to the limited number of variables investigated, which potentially limits the validity of results; this aspect has already been explained previously. We do believe that a considerable number of other variables could be considered, not only to strengthen the correlations we have highlighted, but also to characterize new ones. Variables such as HPV vaccination status, HPV genotyping, and vaginal microbiota could possibly be included in the risk stratification process, once definitely validated as clinically meaningful. It would also be desirable to extend this study to other referral institutions with different patient cohorts, with the goal of testing the validity of the proposed model.

A further limitation of our study is the length of follow-up considered: a larger amount of data would be advisable to evaluate the effectiveness of the proposed model in a longer follow-up period. However, as stated in the study design, limiting the investigation to the first two years of follow-up was chosen in consideration of the highest risk of disease recurrence in the first 24 months after treatment [18,51], and as a function of a correct dataset for a proper statistical analysis.

## 5. Conclusions

Women who have been treated for High-Grade Cervical Intraepithelial Neoplasia are at increased risk of recurrent disease and progression to cervical cancer and therefore require a careful and personalized follow-up to avoid the potential negative consequences. The results of the present study provide insights that can contribute to the practical realization of this goal. The engineered algorithm has proven to be a simple, reproducible, and cost-effective tool that could be usefully introduced into clinical practice in any setting.

In conclusion, investigating the relative prognostic weight of surgical margins status, hrHPV persistence, the neutrophil–lymphocyte ratio, and their possible associations, has demonstrated the feasibility of developing a mathematical/statistical model for the risk-based stratification of patients at different risks of recurrence after surgical treatment for High-Grade Cervical Intraepithelial Neoplasia (HG-CIN).

## Figures and Tables

**Table 1:** Combinations of input values for the three predictors.

A_1_	A_2_	A_3_
1	1	1
1	1	0
1	0	1
0	1	1
1	0	0
0	1	0
0	0	1
0	0	0

**Table 2:** Demographic and clinical-pathological records of study cohort.

**Patient Age**	min	18	
	max	73	
	mean	38.4	
	median	37.5	
**Cone Histology**	CIN1	39 (9.1%)	
	CIN2	192 (44.9%)	
	CIN3	162 (37.9%)	
	CIS	33 (7.7%)	
	G-CIN2	1 (0.2%)	
	G-CIN3	1 (0.2%)	
**Post-Treatment hrHPV**	positive	115 (27%)	
	negative	313 (73%)	
**Surgical Margins**	positive	65 (15.2%)	
	negative	363 (84.8%)	
**Recurrences**	yes	86 (20.1%)	at 12 months: 78 (90.6%)
			at 24 months: 8 (9.4%)
	no	342 (79.9%)	

**Table 3:** Recurrence risk related to variables—Univariate logistic regression.

Variables	*p*-Value	OR	95% CI	
**NLR ≥ 2 + HPV persistence**	0.000	3.417	1.733	6.737
**NLR ≥ 2 + Margins**	0.000	4.507	2.006	10.126
**HPV persistence + Margins**	0.000	6.913	2.202	21.705
**NLR ≥ 2 + HPV persistence + Margins**	0.027	5.512	1.210	25.109
**No Risk Factors**	0.000	0.243	0.127	0.463

**Table 4:** Recurrence risk ranking according to variables (non-linear multivariate regression) (*p* < 0.05).

Margins	hrHPV	NLR ≥ 2	% Recurrence Risk	
+	+	+	69.42	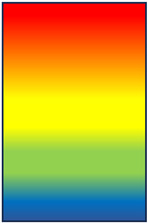
+	+	−	53.34	
+	−	+	42.84	
−	+	+	36.79	
+	−	−	27.69	
−	+	−	22.99	
−	−	+	19.35	
−	−	−	7.48	

## Data Availability

Any request for additional information or data regarding the published article can be obtained through the corresponding author (M.O.).

## References

[1] Gray L.A., Barnes M.L., Lee J.J. (1960). Carcinoma-in-situ and Dysplasia of the Cervix. Ann. Surg..

[2] Antoine T. (1954). Early diagnosis of cancer of the cervix. Am. J. Obstet. Gynecol..

[3] Rawson A.J., Knoblich R. (1957). A Clinicopathologic Study of 56 Cases Showing Atypical Epithelial Changes of the Cervix Uteri. Am. J. Obstet. Gynecol..

[4] Petersen O. (1956). Spontaneous course of cervical precancerous conditions. Am. J. Obstet. Gynecol..

[5] Richart R.M. (1967). Natural History of Cervical Intraepithelial Neoplasia. Clin. Obstet. Gynecol..

[6] Eifel P.J., Klopp A.H., Berek J.S., Konstantinopoulos P.A. (2018). Cancer of the cervix, vagina, and vulva. DeVita, Hellman, and Rosenberg’s Cancer: Principles & Practice of Oncology.

[7] Ferlay J., Soerjomataram I., Dikshit R., Eser S., Mathers C., Rebelo M., Parkin D.M., Forman D., Bray F. (2015). Cancer incidence and mortality worldwide: Sources, methods, and major patterns in GLOBOCAN 2012. Int. J. Cancer..

[8] Arbyn M., Weiderpass E., Bruni L., de Sanjosé S., Saraiya M., Ferlay J., Bray F. (2020). Estimates of incidence and mortality of cervical cancer in 2018: A worldwide analysis. Lancet Glob. Health.

[9] AIRT Working Group I Tumori in Italia—Rapporto 2006. https://www.registri-tumori.it.

[10] Holowaty P., Miller A.B., Rohan T., To T. (1999). Natural History of Dysplasia of the Uterine Cervix. JNCI J. Natl. Cancer Inst..

[11] Babes A. (1928). Diagnostic du cancer du col uterin par les frottis. Presse Méd..

[12] Doorbar J. (2005). The papillomavirus life cycle. J. Clin. Virol..

[13] zur Hausen H. (1977). Human Papillomaviruses and Their Possible Role in Squamous Cell Carcinomas. Curr. Top. Microbiol. Immunol..

[14] Dürst M., Gissmann L., Ikenberg H., zur Hausen H. (1983). Papillomavirus DNA from a cervical carcinoma and its prevalence in cancer biopsy samples from different geographic regions. Proc. Natl. Acad. Sci. USA.

[15] Martin-Hirsch P.P., Paraskevaidis E., Bryant A., Dickinson H.O. (2013). Surgery for cervical intraepithelial neoplasia. Cochrane Database Syst. Rev..

[16] Santesso N., Mustafa R.A., Wiercioch W., Kehar R., Gandhi S., Chen Y., Cheung A., Hopkins J., Khatib R., Ma B. (2016). Systematic reviews and meta- analyses of benefits and harms of cryotherapy, LEEP, and cold knife conization to treat cervical intraepithelial neoplasia. Int. J. Gynecol. Obstet..

[17] Castle P.E., Murokora D., Perez C., Alvarez M., Quek S.C., Campbell C. (2017). Treatment of cervical intraepithelial lesions. Int. J. Gynecol. Obstet..

[18] Kocken M., Helmerhorst T.J., Berkhof J., Louwers J.A., Nobbenhuis M.A.E., Bais A.G., Hogewoning C.J.A., Zaal A., Verheijen R.H.M., Snijders P.J.F. (2011). Risk of recurrent high-grade cervical intraepithelial neoplasia after successful treatment: A long-term multi-cohort study. Lancet Oncol..

[19] Simões R.B., Campaner A.B. (2013). Post-cervical conization outcomes in patients with high-grade intraepithelial lesions. APMIS.

[20] Lu C.H., Liu F.S., Kuo C.J., Chang C.C., Ho E.S.C. (2006). Prediction of Persistence or Recurrence After Conization for Cervical Intraepithelial Neoplasia III. Obstet. Gynecol..

[21] Bogani G., Di Donato V., Sopracordevole F., Ciavattini A., Ghelardi A., Lopez S., Simoncini T., Plotti F., Casarin J., Serati M. (2020). Recurrence rate after loop electrosurgical excision procedure (LEEP) and laser Conization: A 5-year follow-up study. Gynecol. Oncol..

[22] Hoffman S.R., Le T., Lockhart A., Sanusi A., Dal Santo L., Davis M., McKinney D.A., Brown M., Poole C., Willame C. (2017). Patterns of persistent HPV infection after treatment for cervical intraepithelial neoplasia (CIN): A systematic review. Int. J. Cancer..

[23] Bogani G., Pinelli C., Chiappa V., Martinelli F., Lopez S., Ditto A., Raspagliesi F. (2020). Age-specific predictors of cervical dysplasia recurrence after primary conization: Analysis of 3212 women. J. Gynecol. Oncol..

[24] Mariani L., Sandri M.T., Preti M., Origoni M., Costa S., Cristoforoni P., Bottari F., Sideri M. (2016). HPV-Testing in Follow-up of Patients Treated for CIN2+ Lesions. J. Cancer.

[25] Andersson S., Megyessi D., Belkić K., Alder S., Östensson E., Mints M. (2021). Age, margin status, high-risk human papillomavirus and cytology independently predict recurrent high-grade cervical intraepithelial neoplasia up to 6 years after treatment. Oncol. Lett..

[26] Oliveira C.A.D., Russomano F.B., Gomes Júnior S.C.D.S., Corrêa F.D.M. (2012). Risk of persistent high-grade squamous intraepithelial lesion after electrosurgical excisional treatment with positive margins: A meta-analysis. Sao Paulo Med. J..

[27] Onuki M., Matsumoto K., Sakurai M., Ochi H., Minaguchi T., Satoh T., Yoshikawa H. (2016). Posttreatment human papillomavirus testing for residual or recurrent high-grade cervical intraepithelial neoplasia: A pooled analysis. J. Gynecol. Oncol..

[28] Cecchini S., Carozzi F., Confortini M., Zappa M., Ciatto S. (2004). Persistent Human Papilloma Virus Infection as an Indicator of Risk of Recurrence of High-Grade Cervical Intraepithelial Neoplasia Treated by the Loop Electrosurgical Excision Procedure. Tumori J..

[29] Katki H.A., Schiffman M., Castle P.E., Fetterman B., Poitras N.E., Lorey T., Cheung L.C., Raine-Bennett T., Gage J.C., Kinney W.K. (2013). Five-Year Risk of Recurrence After Treatment of CIN 2, CIN 3, or AIS. J. Low Genit. Tract Dis..

[30] Bruhn L.V., Hyldig N., Schledermann D. (2022). HPV Test as Test of Cure After Conization for CIN2+: A Nationwide Register-Based Cohort Study. J. Low. Genit. Tract Dis..

[31] Kreimer A.R., Schiffman M., Herrero R., Hildesheim A., González P., Burk R.D., Porras C., Sherman M.E., Demuth F., Cheung L. (2012). Long-term risk of recurrent cervical human papillomavirus infection and precancer and cancer following excisional treatment. Int. J. Cancer.

[32] Swift B.E., Wang L., Jembere N., Kupets R. (2020). Risk of Recurrence After Treatment for Cervical Intraepithelial Neoplasia 3 and Adenocarcinoma in Situ of the Cervix: Recurrence of CIN 3 and AIS of Cervix. J. Low. Genit. Tract Dis..

[33] Serati M., Siesto G., Carollo S., Formenti G., Riva C., Cromi A., Ghezzi F. (2012). Risk factors for cervical intraepithelial neoplasia recurrence after conization: A 10-year study. Eur. J. Obstet. Gynecol. Reprod. Biol..

[34] Sand F., Frederiksen K., Kjaer S.K. (2022). Risk of recurrent disease following conization of cervical intraepithelial neoplasia grade 3 according to post-conization HPV status and surgical margins. Gynecol. Oncol..

[35] Farzaneh F., Faghih N., Hosseini M.S., Arab M., Ashrafganjoei T., Bahman A. (2019). Evaluation of Neutrophil–Lymphocyte Ratio as a Prognostic Factor in Cervical Intraepithelial Neoplasia Recurrence. Asian Pac. J. Cancer Prev..

[36] Origoni M., Cantatore F., Candotti G., Candiani M. (2022). Prognostic Significance of Neutrophil/Lymphocytes Ratio (NLR) in Predicting Recurrence of Cervical Dysplasia. BioMed Res. Int..

[37] Templeton A.J., McNamara M.G., Šeruga B., Vera-Badillo F.E., Aneja P., Ocaña A., Leibowitz-Amit R., Sonpavde G., Knox J.J., Tran B. (2014). Prognostic Role of Neutrophil- to-Lymphocyte Ratio in Solid Tumors: A Systematic Review and Meta-Analysis. JNCI J. Natl. Cancer Inst..

[38] Mizunuma M., Yokoyama Y., Futagami M., Aoki M., Takai Y., Mizunuma H. (2015). The pretreatment neutrophil-to-lymphocyte ratio predicts therapeutic response to radiation therapy and concurrent chemoradiation therapy in uterine cervical cancer. Int. J. Clin. Oncol..

[39] Ethier J.L., Desautels D.N., Templeton A.J., Oza A., Amir E., Lheureux S. (2017). Is the neutrophil-to-lymphocyte ratio prognostic of survival outcomes in gynecologic cancers? A systematic review and meta-analysis. Gynecol. Oncol..

[40] Wang L., Jia J., Lin L., Guo J., Ye X., Zheng X., Chen Y. (2017). Predictive value of hematological markers of systemic inflammation for managing cervical cancer. Oncotarget.

[41] Bottari F., Iacobone A.D., Passerini R., Preti E.P., Sandri M.T., Cocuzza C.E., Gary D.S., Andrews J.C. (2019). Human Papillomavirus Genotyping Compared With a Qualitative High-Risk Human Papillomavirus Test After Treatment of High-Grade Cervical Intraepithelial Neoplasia: A Systematic Review. Obstet. Gynecol..

[42] Iacobone A.D., Radice D., Sandri M.T., Preti E.P., Guerrieri M.E., Vidal Urbinati A.M., Pino I., Franchi D., Passerini R., Bottari F. (2021). Human papillomavirus same genotype persistence and risk of cervical intraepithelial neoplasia2+ recurrence. Cancers.

[43] Melnikow J., McGahan C., Sawaya G.F., Ehlen T., Coldman A. (2009). Cervical intraepithelial neoplasia outcomes after treatment: Long-term follow-up from the British Columbia Cohort Study. J. Natl. Cancer Inst..

[44] Dominoni M., Barcellini A., Pasquali M.F., De Silvestri A., Ferretti V.V., Cesari S., Fiandrino G., Orlandi E., Gardella B. (2024). The Role of Neutrophil-Lymphocytes Ratio in the Prognosis of CIN2+ Recurrence after Excisional Treatment. Gynecol. Obstet. Investig..

[45] Herrera-Gomez A., Porter R.M. (2017). Mixed linear-nonlinear least squares regression. arXiv.

[46] Kyrgiou M., Mitra A., Arbyn M., Stasinou S.M., Martin-Hirsch P., Bennett P., Paraskevaidis E. (2014). Fertility, and early pregnancy outcomes after treatment for cervical intraepithelial neoplasia: Systematic review and meta-analysis. BMJ.

[47] Kyrgiou M., Mitra A., Arbyn M., Paraskevaidi M., Athanasiou A., Martin-Hirsch P.P., Bennett P., Paraskevaidis E. (2015). Fertility, and early pregnancy outcomes after conservative treatment for cervical intraepithelial neoplasia. Cochrane Database Syst. Rev..

[48] Arbyn M., Redman C.W.E., Verdoodt F., Kyrgiou M., Tzafetas M., Ghaem-Maghami S., Petry K.U., Leeson S., Bergeron C., Nieminen P. (2017). Incomplete excision of cervical precancer as a predictor of treatment failure: A systematic review and meta- analysis. Lancet Oncol..

[49] Ghaem-Maghami S., Sagi S., Majeed G., Soutter W.P. (2007). Incomplete excision of cervical intraepithelial neoplasia and risk of treatment failure: A meta-analysis. Lancet Oncol..

[50] Bonde J., Bottari F., Iacobone A.D., Cocuzza C.E., Sandri M.T., Bogliatto F., Khan K.S., Ejegod D.M., Gary D.S., Andrews J.C. (2021). Human Papillomavirus Same Genotype Persistence and Risk: A Systematic Review. J. Low. Genit. Tract Dis..

[51] Kechagias K.S., Kalliala I., Bowden S.J., Athanasiou A., Paraskevaidi M., Paraskevaidis E., Dillner J., Nieminen P., Strander B., Sasieni P. (2022). Role of human papillomavirus (HPV) vaccination on HPV infection and recurrence of HPV related disease after local surgical treatment: Systematic review and meta-analysis. BMJ.

[52] Mitra A., MacIntyre D.A., Marchesi J.R., Lee Y.S., Bennett P.R., Kyrgiou M. (2016). The vaginal microbiota, human papillomavirus infection and cervical intraepithelial neoplasia: What do we know and where are we going next?. Microbiome.

[53] Yang Z., Zhang Y., Stubbe-Espejel A., Zhao Y., Liu M., Li J., Zhao Y., Tong G., Liu N., Qi L. (2022). Vaginal microbiota and personal risk factors associated with HPV status conversion—A new approach to reduce the risk of cervical cancer?. PLoS ONE.

[54] Kyrgiou M., Moscicki A.B. (2022). Vaginal microbiome and cervical cancer. Semin. Cancer Biol..

[55] Li X., Xiang F., Liu T., Chen Z., Zhang M., Li J., Kang X., Wu R. (2024). Leveraging existing 16S rRNA gene surveys to decipher microbial signatures and dysbiosis in cervical carcinogenesis. Sci. Rep..

[56] Bogani G., Lalli L., Sopracordevole F., Ciavattini A., Ghelardi A., Simoncini T., Plotti F., Casarin J., Serati M., Pinelli C. (2022). Development of a Nomogram Predicting the Risk of Persistence/Recurrence of Cervical Dysplasia. Vaccines.

[57] Zahorec R. (2021). Neutrophil-to-lymphocyte ratio, past, present and future perspectives. Bratisl. Lek. Listy..

[58] Zou P., Yang E., Li Z. (2020). Neutrophil-to-lymphocyte ratio is an independent predictor for survival outcomes in cervical cancer: A systematic review and meta-analysis. Sci. Rep..

[59] Chun S., Shin K., Kim K.H., Kim H.Y., Eo W., Lee J.Y., Namkung J., Kwon S.H., Koh S.B., Kim H.B. (2017). The Neutrophil-Lymphocyte Ratio Predicts Recurrence of Cervical Intraepithelial Neoplasia. J. Cancer.

[60] Hajizadeh N., Baghestani A.R., Pourhoseingholi M.A., Khadem Maboudi A.A., Farzaneh F., Faghih N. (2021). Evaluation of the Factors Affecting the Cure Rate of Cervical Intra-Epithelial Neoplasia Recurrence Using Defective Models. J. Res. Health Sci..

[61] Ghaem-Maghami S., De-Silva D., Tipples M., Lam S., Perryman K., Soutter W. (2011). Determinants of success in treating cervical intraepithelial neoplasia. BJOG.

[62] Linee Guida Condivise per la Prevenzione del Carcinoma Della Cervice Uterina Raccomandazioni per la Gestione Delle Donne in Follow-Up Post Trattamento per CIN2 e CIN3 Raccomandazioni Pubblicate nel Sistema Nazionale Linee Guida Roma, 14 Ottobre 2021—ISCi in Collaborazione con AIO, AOGOI, SIAPEC-IAV, SICi, SICPCV, SIGO, SItI, SIV-ISV. https://gisci.it/documenti/linee-guida/LG_197_GISCi_Biomarcatori-screening-cervicale_29ago24.pdf.

[63] Rossi P.G., Ricciardi A., Cohet C., Palazzo F., Furnari G., Valle S., Largeron N., Federici A. (2009). Epidemiology and costs of cervical cancer screening and cervical dysplasia in Italy. BMC Public Health.

